# Association Between Weight-Adjusted Waist Index and Emphysema in Adults in the United States: A Cross-Sectional Study Involving 44,949 Participants

**DOI:** 10.3390/arm92060043

**Published:** 2024-11-21

**Authors:** Hui Cheng, Ziheng Yang, Jiateng Guo, Yukun Zu, Fan Li, Bo Zhao

**Affiliations:** Department of Thoracic Surgery, Tongji Hospital, Tongji Medical College, Huazhong University of Science and Technology, Wuhan 430030, China; ch939741609@163.com (H.C.); yzhhbu@126.com (Z.Y.); 18737552580@163.com (J.G.); silentgarfield@163.com (Y.Z.); tjhtsdrli@163.com (F.L.)

**Keywords:** chronic obstructive pulmonary disease (COPD), cross-sectional study, emphysema, NHANES, visceral fat, Weight-Adjusted Waist Index

## Abstract

**Highlights:**

**What are the main findings?**

- Individuals with higher WWI values have a higher risk of emphysema, and it reveals the impact of visceral fat on lung health.

**What are the implications of the main findings?**

- The implication of the main findings: The results of the study highlight the importance of using WWI in clinical settings to assess the risk of emphysema, especially for patients with significant visceral fat accumulation.
- WWI can be incorporated into screening programs and guidelines for respiratory disease prevention, providing a basis for personalized risk assessment and management.

**Abstract:**

**Background**: The relationship between obesity and respiratory diseases has been widely explored. In this context, the Weight-Adjusted Waist Index (WWI) has emerged as a novel metric for assessing visceral fat. This study aims to evaluate the association between WWI and the risk of emphysema in the U.S. population, by utilizing data from the 2001–2018 National Health and Nutrition Examination Survey (NHANES). **Methods**: A cross-sectional study was conducted using NHANES data from 2001 to 2018. Logistic regression models were applied to assess the relationship between WWI and emphysema risk. Interaction and subgroup analyses were performed to explore effect modifiers. **Results**: Our study included a total of 44,949 American adults. The results of the multivariable logistic regression analysis revealed an association between WWI and the incidence of emphysema. In the fully adjusted model, the probability of developing emphysema was 1.5 times higher in the group with WWI > 10.46 compared to those with WWI ≤ 10.46, with an odds ratio of [1.5 (1.1, 1.9), *p* = 0.003]. Subgroup analysis showed stronger associations among males, non-Hispanic Whites, and individuals with hypertension. Furthermore, we used a two-piece linear regression model and found a nonlinear association between WWI and emphysema, with a breakpoint at 12.5. **Conclusions**: Our findings indicate a significant association between WWI levels and emphysema. Larger-scale prospective studies are needed to further explore the role of WWI in emphysema.

## 1. Introduction

Emphysema is a chronic respiratory disease characterized by irreversible damage to the alveolar walls and abnormal expansion of lung tissue. It represents an important component of Chronic Obstructive Pulmonary Disease (COPD) [1]. Key risk factors for emphysema include smoking, environmental pollution, and genetic predisposition. These factors contribute to a decline in respiratory function and quality of life, making emphysema a major global public health challenge [2].

In parallel, obesity, particularly visceral fat accumulation, is an important risk factor for various chronic diseases such as cardiovascular disease, type 2 diabetes, and metabolic syndrome [3,4]. Although Body Mass Index (BMI), is widely used to assess obesity, it has limitations in evaluating visceral fat distribution [5]. While Dual-Energy X-ray Absorptiometry (DEXA) is highly accurate for assessing body composition, including visceral fat distribution, it has limitations such as high cost, limited accessibility, and the requirement for specialized equipment [6]. In contrast, WWI calculates the ratio of waist circumference to the square root of weight, effectively controlling for the effect of weight on waist circumference and thus providing a more reliable measure of visceral obesity. Thus, the Weight-Adjusted Waist Index (WWI), as a new obesity assessment measure, more accurately reflects an individual’s visceral fat mass and is closely related to various metabolic risks [7].

Multiple studies have confirmed the significant association between WWI and chronic diseases. For example, higher WWI is significantly associated with an increased risk of cardiovascular disease, diabetes, and chronic kidney disease [8,9], Nevertheless, the relationship between WWI and emphysema has not been thoroughly studied. Given that visceral fat may affect lung function through mechanisms such as inflammatory responses and oxidative stress [10], there is a strong need to explore the potential link between WWI and emphysema to understand the impact of obesity on lung health.

Thus, this study aims to use the National Health and Nutrition Examination Survey (NHANES) database to evaluate the association between WWI and emphysema. This study uses a cross-sectional design, aiming to provide new insights into the potential role of WWI as a predictor of emphysema and to lay the foundation for future longitudinal research.

## 2. Materials and Methods

### 2.1. Survey Description

This study is based on data from the 2001–2018 NHANES in the United States. NHANES, conducted by the National Center for Health Statistics (NCHS), uses a multistage stratified random sampling design that aims to assess the health and nutritional status of the non-institutionalized U.S. population. The survey covers a broad spectrum of biometric measurements, laboratory tests, and health questionnaire data, offering national representativeness. Each participant signed an informed consent form, and the study protocol was approved by the NCHS Research Ethics Review Board.

### 2.2. Study Population

**Figure 1:**
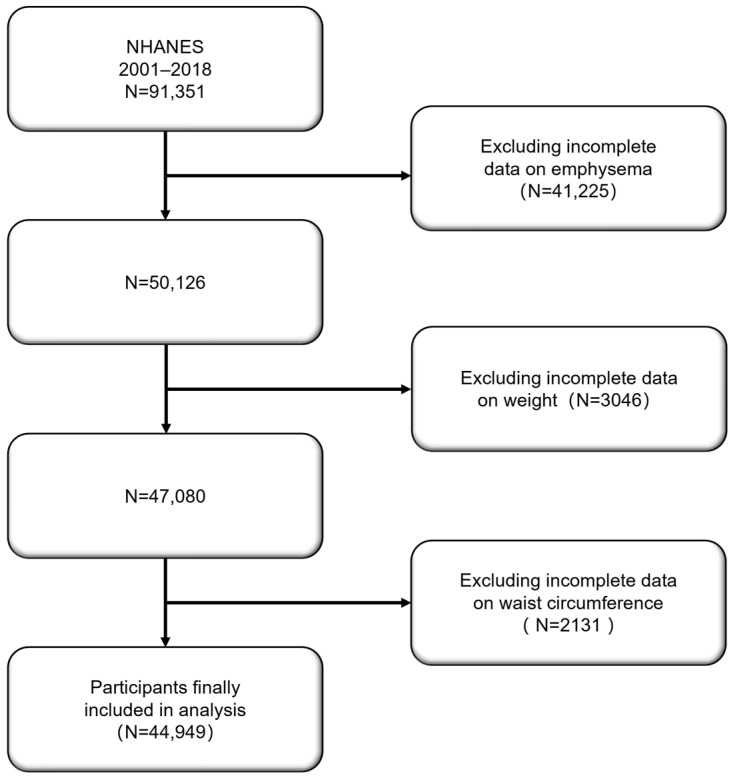
Flowchart of study selection.

The study population consisted of adults (aged ≥ 20 years) who participated in the NHANES survey between 2001 and 2018. A total of 91,351 participants were initially included in the study. During data cleaning, individuals who did not answer the question “Ever told you had emphysema?” were excluded, leading to the exclusion of 41,225 participants. In further screening, participants lacking weight (*n* = 3046) or waist circumference (*n* = 2131) data were excluded. Ultimately, 44,949 eligible participants were included in the analysis for this study (Figure 1).

### 2.3. Assessment of Emphysema and Weight-Adjusted Waist Index

The assessment of emphysema was based on a self-reported question in the NHANES questionnaire entitled “Ever told you had emphysema?”. Individuals who answered “yes” were defined as having emphysema. As for WWI, the calculation formula is WWI = Waist Circumference (cm)/Square Root of Weight (kg^0.5^). Studies have found that 10.46 cm/√kg is considered a critical threshold for WWI. Below this value, WWI is negatively associated with mortality. However, when WWI exceeds this value, the risk of mortality increases significantly [11]. Therefore, WWI is categorized into two groups, the low WWI group (G1: WWI ≤ 10.46) and the high WWI group (G2: WWI > 10.46), for further analysis.

To control for potential confounding factors that may influence the association between WWI and emphysema, a variety of covariates were included in this study. The final covariates included age, gender, race (Mexican American/Other Hispanic/Non-Hispanic White/Non-Hispanic Black/Other Race), education level (less than high school/high school/more than high school), marital status, family income (USD 0 to USD 24,999/USD 25,000 to USD 74,999/USD 75,000 and over), smoking status (whether smoked more than 100 cigarettes in a lifetime), BMI, diabetes (whether diagnosed with diabetes), hypertension (whether diagnosed with hypertension), and family income-to-poverty ratio (PIR). Multiple imputation was used for missing continuous variables, and proportional imputation was applied to categorical variables.

### 2.4. Statistical Analysis

The statistical analysis of this study primarily included the following steps: descriptive statistics were performed on the baseline characteristics of the participants included in the analysis. Hypothesis testing was used to analyze whether the data conform to a normal distribution. The Pearson correlation coefficient analysis was used to assess the linear relationship between WWI and emphysema. For continuous variables (e.g., age, PIR), means and standard deviations (SD) were used to describe them, and differences between WWI groups were compared using t-tests. For categorical variables (e.g., gender, race, marital status), percentages were used for description, and group differences were compared using the chi-square tests. All statistical analyses utilized the sample weights provided by NHANES to ensure the national representativeness of the results.

To evaluate the association between WWI and emphysema, multivariate logistics regression models were used to calculate the odds ratios (OR) and 95% confidence intervals (CI) for emphysema at different WWI levels.

The analysis included the following three models:

- Model 1: Unadjusted model, which evaluated the raw association between WWI and emphysema;
- Model 2: Partially adjusted model, adjusting for potential confounders such as age, gender, and race;
- Model 3: Fully adjusted model, further adjusting for variables such as education level, marital status, household income, smoking status, hypertension, BMI, and family income-to-poverty ratio (PIR).

All regression analysis results were evaluated for statistical significance using *p*-values < 0.05.

To determine whether the association between WWI and emphysema is consistent across different population characteristics, extensive subgroup analyses and interaction tests were conducted. The subgroup analysis was stratified by variables such as age, gender, race, education level, marital status, household income, diabetes, hypertension, and smoking status.

Smoothing curve fitting was used to explore the nonlinear relationship between WWI and emphysema. By incorporating WWI as a continuous variable into the regression model, segmented linear regression was used to evaluate potential nonlinear associations and identify key breakpoints. Threshold effect analysis further examined the impact of WWI on emphysema risk: OR values and 95% CIs were calculated separately for WWI below and above the threshold.

All statistical analyses were performed using R software (version 4.1.3) and the EmpowerStats platform (version 4.2), with two-sided *p*-values < 0.05 considered statistically significant.

## 3. Results

### 3.1. Baseline Characteristics of the Study Population

Table 1 summarizes the baseline characteristics of the study participants grouped by WWI. A total of 44,949 participants were included in the analysis, of which 876 had emphysema, with a prevalence rate of 1.95%. The Pearson correlation coefficient between WWI and emphysema is r = 0.1 (*p* < 0.001). All participants were divided into the low WWI group (G1: WWI ≤ 10.46, *n* = 11,178) and the high WWI group (G2: WWI > 10.46, *n* = 33,771). Participants in the high WWI group had a significantly higher mean age than those in the low WWI group (52.9 years vs. 37.8 years, *p* < 0.001). There was also a significant difference in gender distribution, with a higher proportion of women in the high WWI group (54.8% vs. 41.6%, *p* < 0.001). In terms of racial distribution, the proportion of non-Hispanic Whites was similar between the two groups (G1: 44.1% vs. G2: 43.9%), while non-Hispanic Blacks had a significantly higher proportion in the low WWI group (29.3% vs. 18.4%, *p* < 0.001). Education level also varied significantly, with a higher proportion of participants in the low WWI group having more than a high school education compared to the high WWI group (62.2% vs. 47.4%, *p* < 0.001). Furthermore, marital status and household income differed significantly between the two groups, with a higher proportion of unmarried individuals in the low WWI group, while the proportion of middle-income families (USD 25,000 to USD 74,999) was slightly higher in the high WWI group. Regarding health conditions, the high WWI group had a higher prevalence of diabetes (15.5% vs. 3.0%, *p* < 0.001) and hypertension (40.8% vs. 15.6%, *p* < 0.001). Additionally, smoking behavior differed significantly, with a higher proportion of former smokers in the high WWI group (46.4% vs. 42.1%, *p* < 0.001). Notably, the prevalence of emphysema in the high WWI group was 2.4%, significantly higher than the 0.7% in the low WWI group (*p* < 0.001).

### 3.2. Multivariable Logistic Regression Results

Table 2 presents the multivariable logistic regression results of the association between WWI and emphysema using NHANES data from 2001–2018.

Three models were used to evaluate this relationship, as follows:

- Model 1 (unadjusted) demonstrated that WWI, as a continuous variable, was significantly associated with a higher likelihood of emphysema (OR: 2.1, 95% CI: 1.9–2.3);
- Model 2 (partially adjusted) accounted for basic demographic variables such as age, gender, and race. Even after these adjustments, the association between WWI and emphysema remained significant (OR: 1.7, 95% CI: 1.5–1.8);
- Model 3 (fully adjusted) controlled for additional confounders such as education level, marital status, household income, smoking status, diabetes, hypertension, BMI, and PIR. Even in this model, the association between WWI and emphysema remained significant (OR: 1.4, 95% CI: 1.2–1.5), with each unit increase in WWI raising the likelihood of emphysema by 40%. The prevalence rate of emphysema in the high WWI group was 1.5 times higher than in the low WWI group (OR: 1.5, 95% CI: 1.1–1.9, *p* for trend = 0.003).

### 3.3. Subgroup Analysis and Interaction Test Results

**Figure 2:**
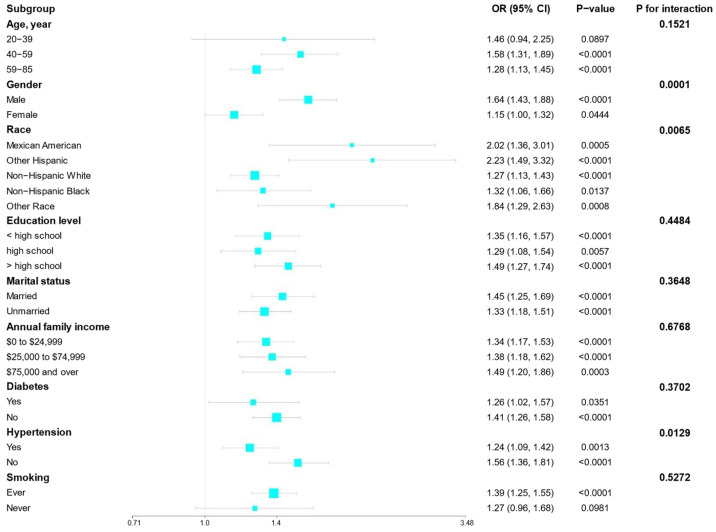
Subgroup analysis of the association between WWI and emphysema using NHANES 2001–2018. Age, gender, race, education level, marital status, diabetes, annual family income, smoking, hypertension, BMI, and PIR were adjusted.

Figure 2 presents the results of the subgroup analysis of the association between WWI and emphysema. In certain demographic and health-related variables, the association between WWI and emphysema was significantly affected by interaction effects. In terms of gender, men (OR: 1.64, 95% CI: 1.43–1.88, *p* < 0.0001) demonstrated a stronger association between WWI and emphysema compared to women (OR: 1.15, 95% CI: 1.00–1.32, *p* = 0.0444), with a *p* for the interaction of 0.0001. The racial subgroup analysis revealed that non-Hispanic Whites (OR: 2.23, 95% CI: 1.49–3.32, *p* < 0.0001) and other races (OR: 1.84, 95% CI: 1.29–2.63, *p* = 0.0008) exhibited a stronger correlation between WWI and emphysema. Conversely, non-Hispanic Blacks (OR: 1.32, 95% CI: 1.06–1.66, *p* = 0.0137) showed a weaker association, with a *p* for the interaction of 0.0065. In the subgroup of individuals with hypertension, those with hypertension (OR: 1.56, 95% CI: 1.36–1.81, *p* < 0.0001) demonstrated a stronger association between WWI and emphysema compared to those without hypertension (OR: 1.24, 95% CI: 1.09–1.42, *p* = 0.0013), with a *p* for the interaction of 0.0129.

Moreover, for the following variables, although the association between WWI and emphysema remained significant across different subgroups, the interaction effects did not show significance: age (*p* for interaction = 0.1521), education level (*p* for interaction = 0.4484), marital status (*p* for interaction = 0.3648), household income (*p* for interaction = 0.6768), diabetes (*p* for interaction = 0.3702), and smoking (*p* for interaction = 0.5272).

### 3.4. Smoothing Curve Fitting and Threshold Effect Analysis

**Figure 3:**
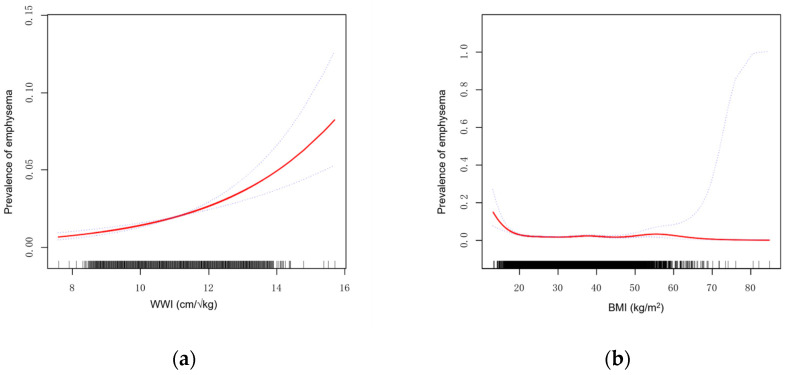
(**a**) Smoothed curve fit plot between WWI and emphysema. (**b**) Smoothed curve fit plot between BMI and emphysema. This is a figure.

This study additionally explored the relationship between BMI and emphysema. Using the same fully adjusted model, Figure 3 illustrates the smoothing curve fitting results of WWI and BMI with emphysema, shown as Figure 3a,b, respectively. In Figure 3a, the relationship between WWI and the prevalence of emphysema is displayed. At lower WWI values (approximately between 8 and 10), the prevalence of emphysema remains low. However, as WWI values increase, particularly beyond 12.5, the prevalence of emphysema rises sharply, indicating a significant positive trend. In contrast, Figure 3b presents the relationship between BMI and the prevalence of emphysema. Compared to WWI, the BMI curve displays a more complex pattern. In the low to moderate BMI range, the prevalence of emphysema remains low, and the curve is relatively flat across most of the BMI spectrum, showing little upward trend. However, at extremely high BMI values (greater than 50), the prevalence of emphysema suddenly spikes, though this signal may be influenced by sample size limitations or other confounding factors.

Therefore, to further explore the relationship between WWI and emphysema, a threshold effect analysis was conducted, as demonstrated in Table 3. First, in the linear model (Model 1), WWI was significantly associated with a positive risk of emphysema, with an OR of 1.4 (95% CI: 1.3, 1.6, *p* < 0.001). In the segmented linear model, the relationship between WWI and emphysema was assumed to be nonlinear, and a key threshold point for WWI was identified at 12.5. This suggests that at WWI = 12.5, the effect of WWI on emphysema prevalence might shift. The results showed that when WWI was less than 12.5, the association with emphysema remained significant (OR = 1.5, 95% CI: 1.4, 1.7, *p* < 0.001). Conversely, when WWI exceeded 12.5, the association weakened and became statistically nonsignificant (OR = 0.8, 95% CI: 0.5, 1.4, *p* = 0.394). The difference between the two segments was statistically significant (*p* = 0.028), and the likelihood ratio test indicated that the threshold effect model provided a better fit to the data than the linear model (*p* = 0.020), as shown in Table 3.

## 4. Discussion

This study is the first to systematically explore the relationship between WWI and emphysema. By utilizing data from 44,949 participants in the 2001–2018 NHANES database and employing multivariable logistic regression models, interaction analysis, and threshold effect analysis, we found a positive association between WWI and emphysema prevalence on the left side of the breakpoint (12.5), while no statistical significance was observed on the right side. This marks the first identification of the nonlinear relationship between WWI, an obesity measure reflecting visceral fat mass, and emphysema. These results enrich the current understanding of the relationship between obesity and lung health and provide a new perspective for further exploration of the role of visceral fat in respiratory diseases.

Previous studies have extensively examined the connection between obesity and lung diseases, particularly chronic obstructive pulmonary disease (COPD) and emphysema. BMI has traditionally been used as the primary measure for assessing obesity, and many studies have confirmed a negative correlation between BMI and emphysema [12,13]. However, BMI has significant limitations: it not only fails to differentiate between fat tissue and lean mass but also cannot accurately represent fat distribution within the body, potentially contributing to substantial differences between individuals and the “obesity paradox” [14]. Dual-Energy X-ray Absorptiometry (DEXA) is another established method used to assess body composition, including the distribution of fat, muscle, and bone. DEXA is highly accurate and can provide detailed information on regional fat deposits, including visceral and subcutaneous fat. However, DEXA has several limitations, such as high cost, limited availability, and the need for specialized equipment and trained personnel, making it impractical for large-scale public health screening or routine clinical use [6]. In contrast, WWI, as a novel index, more conveniently reflects visceral fat accumulation. Studies have shown that WWI is significantly associated with the risk of cardiovascular diseases, diabetes, asthma, and metabolic syndrome [15,16,17,18]. However, the literature is limited on the direct relationship between WWI and lung diseases, especially emphysema. Our study addresses this gap by being the first to reveal a significant association between WWI and emphysema. The results of the smoothing curve fitting suggest that BMI may have limitations in predicting emphysema risk. BMI primarily reflects overall body weight rather than specific fat distribution, particularly visceral fat. Visceral fat has a more direct impact on emphysema, which may explain why the association between BMI and emphysema is weaker and exhibits nonlinear characteristics. In contrast, WWI shows a stronger association with emphysema risk. The significant upward trend in the WWI curve highlights its sensitivity in capturing the impact of visceral fat on emphysema risk, whereas BMI lacks this sensitivity. This difference may arise from WWI’s more accurate reflection of visceral fat distribution, which is closely linked to metabolic diseases and inflammatory responses. When comparing obesity measures and emphysema prevalence, WWI demonstrates clear advantages over BMI.

This study found a significant positive correlation between WWI and emphysema prevalence. This association can be explained by several physiological mechanisms, especially those related to the metabolism and inflammatory effects of visceral fat. Visceral fat is a metabolically active tissue capable of secreting various pro-inflammatory cytokines and hormones, such as tumor necrosis factor-α (TNF-α), interleukin-6 (IL-6), and C-reactive protein (CRP) [19,20,21]. These pro-inflammatory factors affect lung tissue through systemic inflammatory responses, leading to chronic inflammation and oxidative stress [22,23]. Oxidative stress and inflammation are key pathological mechanisms in the development of emphysema, as they damage alveolar structures, destroy elastic fibers, and induce apoptosis of alveolar wall cells, leading to permanent alveolar expansion and loss of lung tissue elasticity [24,25]. Additionally, visceral fat is closely related to insulin resistance, which is considered a potential driver of systemic inflammation [26]. Obesity-related metabolic disorders, such as insulin resistance and lipid metabolism dysregulation, may further exacerbate lung inflammation through complex biological signaling pathways, contributing to the development of emphysema [27].

In the threshold effect analysis, we found that the association between WWI and emphysema significantly weakened when WWI exceeded 12.5. This may reflect multi-level physiological regulatory mechanisms. First, as WWI increases, visceral fat accumulation may reach a critical point of metabolic adaptation [28]. In cases of high visceral fat load, the body may adjust metabolic pathways or activate protective mechanisms, such as anti-inflammatory molecules or antioxidant defense systems, to mitigate the effects of excessive fat accumulation on organs [29,30]. This compensatory mechanism may prevent the risk of emphysema from significantly rising with further increases in WWI in individuals with high WWI. Second, individual differences in fat distribution may also explain this phenomenon. Some individuals with high WWI may have a greater capacity for subcutaneous fat storage, which, compared to visceral fat, has less impact on metabolism and inflammation and may even exert protective effects [31,32]. This heterogeneity in fat distribution could account for the reduced emphysema risk observed in individuals with high WWI. Furthermore, the threshold effect of WWI may also be linked to changes in muscle mass. Obesity not only reflects fat accumulation but may also involve a decline in skeletal muscle mass, particularly in the elderly. Sarcopenia has been shown to correlate with the severity of respiratory diseases [33,34]. Therefore, as WWI exceeds a certain threshold, the effects of muscle loss may offset the contribution of visceral fat to emphysema risk.

Lastly, since the sample size with WWI > 12.5 represents only 4.49% of the total sample, this relatively small sample size may lead to insufficient statistical power. Limited sample sizes can hinder the model’s ability to detect real effects, and even when the effect exists, a small sample size may prevent it from reaching statistical significance. Increasing the sample size, particularly in the high WWI range, to improve detection power could provide further insights into the relationship between high WWI and emphysema prevalence.

The interaction analysis revealed that gender, race, and hypertension status significantly moderated the association between WWI and emphysema risk. The association between WWI and emphysema was more pronounced in men, non-Hispanic Whites, and individuals with hypertension. These interaction results suggest that certain groups may be more sensitive to visceral fat accumulation, with more severe metabolic consequences. First, men generally store more visceral fat than women, and male fat distribution tends toward central obesity, with more fat accumulating in the abdomen [35]. This fat distribution pattern makes men more susceptible to the metabolic consequences of visceral fat, particularly regarding lung health [36,37]. Studies show that higher visceral fat levels in men are closely related to increased systemic inflammation, which exacerbates the development of emphysema [38]. Racial differences may also play a role, influencing visceral fat metabolism through genetic and environmental factors. For instance, non-Hispanic Whites tend to have higher visceral fat stores, while non-Hispanic Blacks and other minority groups may have fat distribution more inclined toward subcutaneous fat [39]. This partially explains why the association between WWI and emphysema is more pronounced in the non-Hispanic White population. Hypertension, as a component of metabolic syndrome, is also closely related to visceral fat accumulation [32]. Individuals with hypertension often exhibit higher levels of visceral fat, further aggravating systemic inflammation and oxidative stress [40]. Therefore, it is unsurprising that the association between WWI and emphysema is more pronounced in individuals with hypertension.

This study systematically explored the association between WWI and emphysema for the first time, revealing that WWI is an independent predictor of emphysema risk. Compared to traditional obesity indicators like BMI, WWI more accurately reflects the impact of visceral fat accumulation on lung health, highlighting the role of visceral fat in the development of emphysema through chronic inflammation and oxidative stress. Although the Pearson correlation between WWI and the prevalence of emphysema is not high, multivariate regression analysis confirmed a significant positive correlation, emphasizing the potential value of controlling WWI in reducing the incidence of emphysema. This study provides a foundation for using WWI to assess the risk of obesity-related respiratory diseases in clinical practice and offers guidance for personalized risk assessments in specific populations. Furthermore, incorporating WWI into routine clinical evaluations has the potential to improve the early detection and management of individuals at higher risk for respiratory complications, particularly in targeted screening programs. The use of WWI could also influence health guidelines, offering a more effective tool for stratifying obesity-related risks and shaping intervention strategies. Additionally, the findings offer new perspectives for future preventive intervention strategies, calling for the widespread use of WWI as an emerging obesity assessment tool in public health and clinical practice and encouraging individuals to be mindful of controlling their WWI.

### 4.1. Strengths

This study utilized the NHANES database, which contains a nationally representative sample spanning various demographic characteristics and health conditions, enhancing the generalizability of the study results. In our analysis, we adjusted for multiple confounding factors, including age, gender, race, education level, household income, marital status, smoking status, diabetes, and hypertension, to ensure the independence and reliability of the association between WWI and emphysema. Interaction tests and subgroup analyses were conducted to reveal the heterogeneity of the relationship between WWI and emphysema across different demographic characteristics and health conditions, allowing for a more nuanced interpretation of the results. This study is also the first to use WWI in the analysis of emphysema. As an emerging obesity assessment metric, the application of WWI in emphysema research opens up new perspectives for future clinical and epidemiological studies.

### 4.2. Limitations

Limitations of the cross-sectional study design. As this study employs a cross-sectional design, it cannot establish a causal relationship between WWI and emphysema. Future longitudinal studies are needed to further explore the causal link between the two. The influence of potential confounders cannot be fully excluded. Although we adjusted for various confounding factors, unmeasured confounders, such as diet and environmental factors, may still be present, potentially having a significant impact on the development of emphysema. Additionally, due to the limitations of the NHANES database, this study relied on self-reported emphysema diagnoses, which may introduce information bias. Moreover, the dynamic changes in WWI and emphysema over time were not captured, limiting the ability to evaluate their long-term relationship. More rigorous randomized controlled trials are required to investigate this issue further.

## 5. Conclusions

Our study highlights a significant positive association between the Weight-Adjusted Waist Index (WWI) and the risk of emphysema among U.S. adults. This association was found to be stronger among specific subgroups, particularly in males, non-Hispanic Whites, and individuals with hypertension. These key findings suggest that age, gender, race, and comorbidities like hypertension can modulate the impact of visceral fat distribution on lung health. These results emphasize the need for targeted prevention strategies in populations more vulnerable to visceral fat-related respiratory complications. Future research should consider longitudinal designs to establish causality and explore mechanisms linking WWI to lung disease pathogenesis.

## Figures and Tables

**Table 1:** Baseline characteristics of participants categorized into two groups based on WWI among adults in NHANES 2001–2018.

Characteristics	Weight-Adjusted Waist Index(cm/√kg)		*p*-Value
	G1 (7.59~10.46) N = 11,178	G2 (10.46~15.70) N = 33,771	
Age, year (SD)	37.8 (14.4)	52.9 (17.5)	<0.001
Gender, %			<0.001
Male	58.4	45.2	
Female	41.6	54.8	
Race, %			<0.001
Mexican American	9.3	19.2	
Other Hispanic	6.2	9.2	
Non-Hispanic White	44.1	43.9	
Non-Hispanic Black	29.3	18.4	
Other Race	11.1	9.3	
Education level, %			<0.001
<high school	16.4	28.8	
high school	21.4	23.8	
>high school	62.2	47.4	
Marital status, %			<0.001
Married	44.2	55.5	
Unmarried	55.8	44.5	
Annual family income, %			<0.001
USD 0 to USD 24,999	29.6	32.2	
USD 25,000 to USD 74,999	38.2	40.0	
USD 75,000 and over	32.2	27.8	
PIR (SD)	2.8 (1.7)	2.5 (1.6)	<0.001
BMI (SD)	24.9 (4.8)	30.3 (6.7)	<0.001
Smoking, %			<0.001
Ever	42.1	46.4	
Never	57.9	53.6	
Diabetes, %			<0.001
Yes	3.0	15.5	
No	97.0	84.5	
Hypertension, %			<0.001
Yes	15.6	40.8	
No	84.4	59.2	
Emphysema, %			<0.001
Yes	0.7	2.4	
No	99.3	97.6	

Abbreviation: PIR, Ratio of family income to poverty, BMI, Body mass index, G, group.

**Table 2:** Associations between WWI and emphysema in NHANES 2009–2014 based on multivariable logistic regression models.

WWI	Model 1	Model 2	Model 3
	OR (95%CI)	OR (95%CI)	OR (95%CI)
Continuous	2.1 (1.9, 2.3)	1.7 (1.5, 1.8)	1.4 (1.2, 1.5)
Categories			
≤10.46	1.0	1.0	1.0
>10.46	3.7 (2.9, 4.7)	1.9 (1.4, 2.4)	1.5 (1.1, 1.9)
*p* for trend	<0.001	<0.001	0.003

**Table 3:** Threshold effect analysis of WWI on emphysema.

	OR (95% CI)	*p*-Value
Model 1 ^a^	1.4 (1.3, 1.6)	<0.001
Model 2 ^b^		
Breakpoint (K)	12.5	
OR1 (<12.5)	1.5 (1.4, 1.7)	**<0.001**
OR2 (>12.5)	0.8 (0.5, 1.4)	0.394
OR2/OR1	0.5 (0.3, 0.9)	**0.028**
Log likelihood ratio	**0.020**	

Significant values are in bold. ^a^ Model 1: Standard linear model. ^b^ Model 2: Two-piecewise linear model. Adjust for age, gender, race, education level, marital status, diabetes, annual family income, smoking, hypertension, BMI, and PIR.

## Data Availability

All NHANES data for this study are publicly available and can be found at https://wwwn.cdc.gov/nchs/nhanes (accessed on 1 September 2024).
